# De novo design of ATPase based on a blueprint optimized for harboring the P‐loop motif

**DOI:** 10.1002/pro.70132

**Published:** 2025-05-13

**Authors:** Takahiro Kosugi, Mikio Tanabe, Nobuyasu Koga

**Affiliations:** ^1^ Research Center of Integrative Molecular Systems Institute for Molecular Science (IMS), National Institutes of Natural Sciences (NINS) Okazaki Aichi Japan; ^2^ Exploratory Research Center on Life and Living Systems (ExCELLS) National Institutes of Natural Sciences (NINS) Okazaki Aichi Japan; ^3^ Molecular Science Program SOKENDAI (The Graduate University for Advanced Studies) Hayama Kanagawa Japan; ^4^ PRESTO Japan Science and Technology Agency Kawaguchi Saitama Japan; ^5^ Structural Biology Research Center Institute of Materials Structure Science, High Energy Accelerator Research Organization (KEK) Tsukuba Japan; ^6^ Advanced Data Science Center for Protein Research (ASPiRE) Institute for Protein Research (IPR), Osaka University Suita Osaka Japan

**Keywords:** ATPase, enzyme, P‐loop, protein design

## Abstract

De novo design of proteins has seen remarkable recent progress and has provided understanding of folding and functional expression. However, rationally creating enzymes with high activity comparable to most naturally occurring enzymes remains challenging. Here, we attempted to design an ATPase de novo, through the exploration of an optimal backbone blueprint to incorporate a conserved phosphate binding motif, the P‐loop, into designed structures. The designed protein, based on the identified blueprint, was found to be a monomer with high thermal stability and exhibited ATPase ability. The crystal structure was closely matched to the design model, both at the overall structure level and within the P‐loop motif. Interestingly, AlphaFold 2 was not able to predict the designed structure accurately, indicating the difficulties of predicting folded structures for novel amino acid sequences. Remarkably, the designed protein exhibited ATPase ability even at temperatures around 100°C, with significantly increased activity. However, the ATPase activity was still not comparable to those of naturally occurring enzymes. This suggests that the P‐loop motif alone is insufficient to achieve the high ATPase activity seen in naturally occurring enzymes, indicating that other structural components—such as a binding pocket optimized for the adenine or ribose moieties of ATP, additional catalytic residues, or structural dynamics that facilitate hydrolysis—are necessary to reach such activity levels.

## 1 INTRODUCTION

De novo protein design provides opportunities to explore fundamental understanding of folding and functional expression programmed into protein structures. A variety of proteins have been designed from scratch, together with the understanding of principles for folding and functional expression (Baker, 2019; Chu et al., 2024; Koga & Koga, 2019; Kortemme, 2024; Woolfson, 2021). However, computationally creating highly active enzymes without further experimental optimizations such as directed evolution or site‐directed mutagenesis remains an unsolved problem (Lovelock et al., 2022; Mak & Siegel, 2014). In this research, we aimed to uncover the design principles to create ATPase with high activity through de novo design, particularly focused on an optimal backbone blueprint, involving secondary structure lengths, loop patterns, and registries between β‐strands, to incorporate a conserved phosphate‐binding motif, known as the P‐loop motif.

**FIGURE 1:**
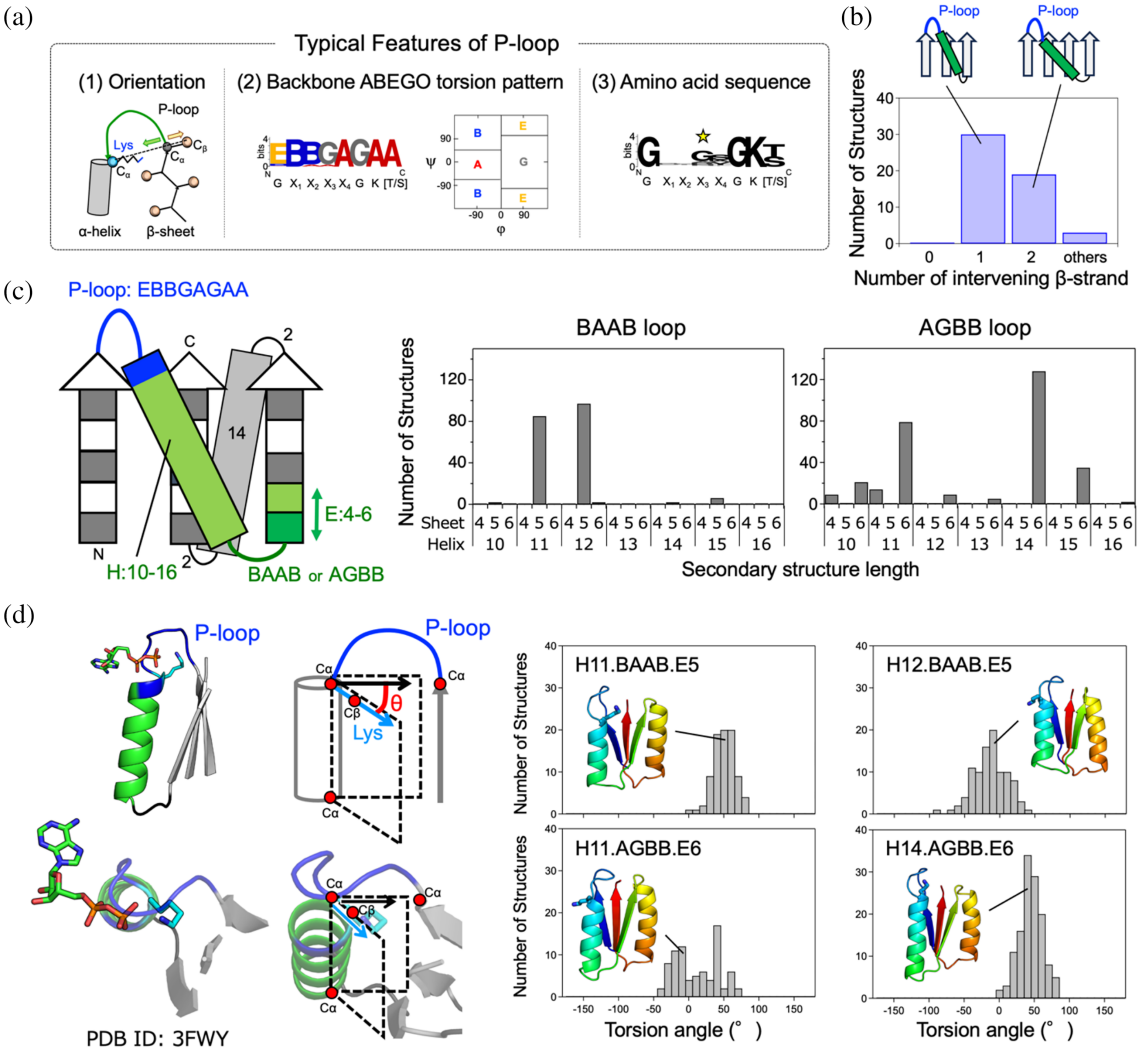
Exploration of the optimal blueprint for de novo design to harbor the P‐loop motif. (a) The typical features of P‐loops observed in naturally occurring proteins (Kosugi et al., 2023). (1) Orientation: the vector from the C_α_ atom of the last strand residue immediately before the P‐loop to the C_α_ atom of the conserved Lys points away from the vector from the C_α_ atom of the same last strand residue to its C_β_ atom. (2) Backbone ABEGO torsion pattern: the backbone conformation of residues in P‐loops typically shows the EBBGAGAA torsion pattern (the torsion A and B are the α‐helix and β‐sheet regions; G and E are the positive ϕ regions). (3) Amino acid sequence: the P‐loop is identified by the conserved sequence GX_1_X_2_X_3_X_4_GK(T/S). (b) The distribution of the number of β‐strands traversed by the β‐(P‐loop)‐α‐β motifs in naturally occurring proteins. (c) Left: explored partial blueprints to identify the optimal one to harbor the P‐loop motif. Right: the distribution of the number of Rosetta folding simulation trajectories, in which the simulated protein, starting from an extended conformation, folded into the structure corresponding to each blueprint. For each blueprint, 500 independent folding simulations were carried out. (d) Left: a typical naturally occurring P‐loop conformation with an ATP molecule. The conserved Lys points inward to the P‐loop cradle to interact with the β‐phosphate moiety of ATP. This structural feature can be captured by considering the orientation angle defined by the following two planes. One plane is defined by the C_α_ and C_β_ atoms of the Lys residue and the C_α_ atom of the last residue of the helix connected to the P‐loop. The other plane is defined by the C_α_ atom of the last β‐strand residue and the C_α_ atoms of the first and last helix residues. Right: the distribution of the number of structures, generated from the above Rosetta folding simulations, with the Lys showing the orientation angles between 40° and 60°.

Many naturally occurring ATPases (Saraste et al., 1990; Walker et al., 1982) possess the P‐loop motif, with a sequence pattern of GX_1_X_2_X_3_X_4_GK[T/S] (X indicates any kind of amino acid residues). Previously, Romero et al. designed proteins with ATPase activity (Romero Romero et al., 2018). In their study, they discovered that inserting the conserved sequence into a loop region of a de novo designed protein scaffold is sufficient to provide ATP hydrolase ability. However, the ATPase activity of designed proteins, approximately one ATP molecule every 30 min, is significantly lower compared to that of naturally occurring enzymes (Mak & Siegel, 2014), prompting further investigation into how to improve catalytic activity. Interestingly, the inserted P‐loop sequence exhibits unstable and dynamic conformations in the NMR structure of the designed protein (PDB 6C2U and 6C2V). The P‐loop motifs in naturally occurring proteins typically show a cradle‐like conformation to grasp the phosphate moieties of the ATP molecule (Figure 1a). We have recently described the characteristic features of the backbone conformation of the P‐loop motif (Figure 1a) (Kosugi et al., 2023). Here, we hypothesize that the low ATPase ability is attributed to the unstable conformation of the inserted P‐loop motif. Based on this hypothesis, we aimed to design ATPases with the P‐loop that adopts the characteristic, stable conformation, by investigating the optimal backbone blueprint for de novo design of proteins harboring the P‐loop motif.

## 2 RESULTS

### 2.1 Exploration of the blueprint optimized for harboring the P‐loop motif

We began by seeking an optimal backbone blueprint, which involves a tertiary arrangement of secondary structures and loop connections (i.e., topology or fold), along with their lengths, to design protein structures that harbor the P‐loop motif. As a first step, we attempted to build a partial blueprint for the structure around an embedded P‐loop motif. The P‐loop motifs are typically located at junctions from a β‐strand to an α‐helix in the α/β class proteins, which are characterized by a β‐sheet sandwiched between α‐helices, composed of repeated β‐α‐β motifs with β‐strands mostly aligned in parallel. Additionally, in the β‐(P‐loop)‐α‐β motif, the first β‐strand starts from the middle of the β‐sheet, and the subsequent α‐helix followed by the second β‐strand traverses one or two β‐strands (Figure 1b). We studied the distribution of the number of β‐strands traversed by β‐(P‐loop)‐α‐β motifs in naturally occurring proteins (Figure 1b). We found that there are no topologies where the first and subsequent β‐strands are adjacent to each other (i.e., the traversed number is zero). In previously designed proteins with the P‐loop motif, this motif was embedded in β‐α‐β motifs where the first and second β‐strands are adjacent to each other (Romero Romero et al., 2018). Therefore, we decided to embed the β‐(P‐loop)‐α‐β motif with one intervening β‐strand (Figure 1c), considering the most common arrangement found in naturally occurring proteins. Furthermore, we previously identified typical local structural features for the P‐loop motif (Kosugi et al., 2023): the vector from the C_α_ atom of the last strand residue immediately before the P‐loop to the C_α_ atom of the conserved Lys points away from the vector from the C_α_ atom of the same last strand residue to its C_β_ atom (Figure 1a, left), and the backbone torsion pattern represented by ABEGO (Wintjens et al., 1996) torsion bins is EBBGAGAA (the torsion bins A and B are the α‐helix and β‐sheet regions; G and E are the positive phi regions) (Figure 1a, middle). With these considerations, we explored the optimal lengths of secondary structures and the connecting loops for the α/β topology shown in Figure 1c by carrying out Rosetta fragment assembly simulations (Simons et al., 1997).

In the Rosetta folding simulations, we fixed the lengths of the first and third β‐strands at five residues and set the loop torsion pattern of the P‐loop motif to EBBGAGAA, as frequently observed in naturally occurring proteins (Figure 1a, middle). The 14‐residue second α‐helix and the two‐residue loops connected to the helix were determined by referring to Tables S1 and S2 in the previous work (Lin et al., 2015). Subsequently, we explored the optimal lengths of the helix connected to the P‐loop motif and the second β‐strand, as well as the backbone torsion patterns, represented by ABEGO torsion bins, of the loop immediately following the helix. Our aim was to identify the helix and β‐strand lengths, as well as loop backbone torsion patterns, that would most frequently fold into the structures designated by the blueprints. For each blueprint, 500 independent folding simulations were carried out, resulting in the identification of four patterns of the helix length, loop ABEGO pattern, and β‐strand lengths: H11.BAAB.E5, H12.BAAB.E5, H11.AGBB.E6, and H14.AGBB.E6 (where H represents an α‐helix; E represents a β‐strand) that consistently folded into the intended blueprint structure.

Next, we sought to identify the most optimal blueprint to harbor the P‐loop among the four identified blueprints by focusing on the side‐chain orientation of the conserved Lys in the P‐loop motif. The P‐loop motif exhibits a cradle‐like conformation that encompasses the α‐ and β‐phosphate groups of ATP, with the conserved Lys primarily interacting with the β‐phosphate moiety (Figure 1d, left). Therefore, we analyzed the four identified patterns to reveal which could exhibit the optimal side‐chain orientation, facilitating the binding of the conserved Lys in the P‐loop motif to the β‐phosphate. To this end, we examined the pattern in which the orientation of the C_α_‐to‐C_β_ vector of the Lys residue points inward toward the P‐loop cradle, relative to the plane defined by the C_α_ atoms of the last strand residue and the first and last helix residues. As a result, the blueprint with a 14‐residue helix, a loop with the AGBB ABEGO torsion pattern, and a six‐residue β‐strand was identified as the most optimal for allowing the Lys residue to point inward to the P‐loop cradle, facilitating the interaction with the β‐phosphate moiety.

**FIGURE 2:**
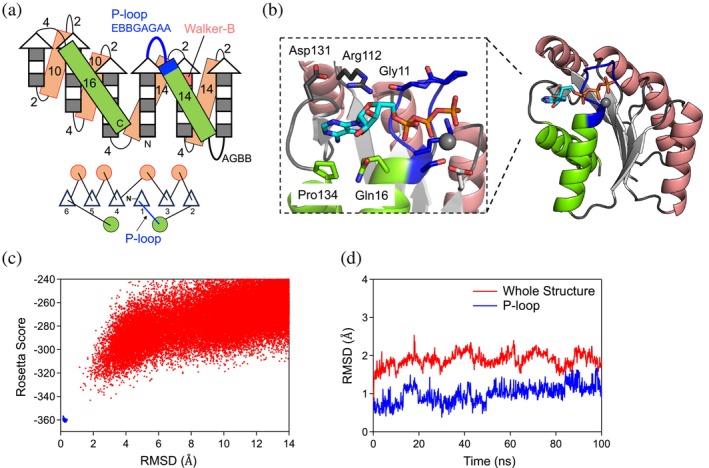
De novo designed protein with the P‐loop motif. (a) The identified optimal backbone blueprint to harbor the P‐loop motif. Strand lengths are represented by filled and empty boxes that indicate pleats coming out and going into the page, respectively. Helices are represented by rectangle boxes. Letter strings next to the loops or on the rectangle boxes indicate their lengths. (b) The designed protein structure based on the optimal blueprint. This structure harboring the P‐loop motif also contains a binding pocket for the base and ribose moieties, allowing it to encompass an entire ATP molecule. (c) The funnel‐shaped energy landscape of the designed protein, PL2x4_2, explored by Rosetta folding simulations. Lowest‐energy structures obtained by 50,000 and 5000 independent Monte Carlo structure prediction trajectories starting from an extended chain (red) and from the design model (blue) are shown. The x and y axes indicate the C_α_ root mean squared deviation (RMSD) from the design model and the Rosetta all‐atom energy, respectively. (d) Conformational fluctuations of PL2x4_2 during MD simulations for the whole structure (red) and the structure of the P‐loop motif (blue). C_α_ RMSD values from the initial structure during the simulations are shown.

Next, we sought to construct a complete blueprint based on the identified partial blueprint, with the aim of the creation of the binding pocket for the adenine base and ribose of ATP. To achieve this, we augmented the partial blueprint by incorporating three additional β‐α‐β motifs (Figure 2a). Register shifts between the fourth and fifth strands, as well as between the fifth and sixth strands, were introduced to facilitate the twisting of the β‐sheet, allowing the C‐terminal helix to interact with the N‐terminal region and the adenine ring of ATP. The lengths of secondary structures and the connecting loops were selected by referencing Tables S1 and S2 in the previous work (Lin et al., 2015), except for the last helix and its connected loop. The length of the last helix was chosen to sufficiently cover the β‐sheet. In our previous de novo designed proteins, loops connecting a β‐strand to an α‐helix were either two or three residues, depending on the helix's orientation relative to the C_α_ to C_β_ vector of the last strand residue (Koga et al., 2012). However, for this design, we opted for a loop length of four residues to provide enhanced conformational freedom to search for conformations in which the loop and following helix can interact with the ATP molecule.

### 2.2 Design of ATPase based on the identified optimal blueprint

Finally, we built protein structures based on the complete backbone blueprint (Figure 2a). We carried out Rosetta fragment assembly simulations, which generated approximately 100 backbone structures harboring the P‐loop motif. For each generated structure, amino acid sequences and their side‐chain conformations were built using RosettaDesign (Leaver‐Fay et al., 2011) to stabilize the entire generated backbone structure as well as to make favorable interactions with the ATP molecule, using multiple ATP conformations and a set of distance constraints used in our previous research (Kosugi et al., 2023). We also attempted to place the Walker‐B motif (i.e., Asp or Glu), which chelates the magnesium ion in coordination with the Thr or Ser residues of the P‐loop motif, at the second‐to‐last residue of the third β‐strand.

The designed structures were filtered based on the binding scores (Rosetta ddG score <−12.0, DSasa score >0.4, and <0.7), the side‐chain packing quality (RosettaHole score <2.0, packstat >0.6) (Sheffler & Baker, 2009), and the sequence‐structure compatibility (Koga et al., 2012). One of the designed structures is shown in Figure 2b. The P‐loop motif binds the phosphate group of the ATP molecule, while the adenine moiety is enclosed by a loop structure leading to the last helix. In this design, PL2x4_2, the adenine ring forms a hydrophobic interaction with Pro134 and two hydrogen bonds with Arg112 and Gln116. Arg112 is further stabilized by two hydrogen bonds with Gly11 in the P‐loop motif and Asp131. Subsequently, we selected designs with funnel‐shaped energy landscapes, explored through Rosetta ab initio structure prediction simulations (Rohl et al., 2004) for the designed structures in the absence of an ATP molecule (Figures 2c and S1). The stability of both the backbone structure and the P‐loop motif was further evaluated by investigating the structural stability during a 100 ns MD simulation (Figures 2d and S1). Finally, three designs that satisfied all of the above criteria were selected for experimental evaluation.

### 2.3 Experimental characterization of the designed ATPases

We obtained synthetic genes encoding the three designs (Table S1). None of the designs showed similarity to known proteins, with a BLAST E‐value <0.001. The proteins were recombinantly expressed in *Escherichia coli* and purified using Ni^2+^‐affinity (Ni‐NTA) chromatography. The purified proteins were characterized using circular dichroism (CD) spectroscopy, size‐exclusion chromatography combined with multi‐angle light scattering (SEC‐MALS), and ^1^H–^15^N heteronuclear single quantum coherence (HSQC) NMR spectroscopy.

**FIGURE 3:**
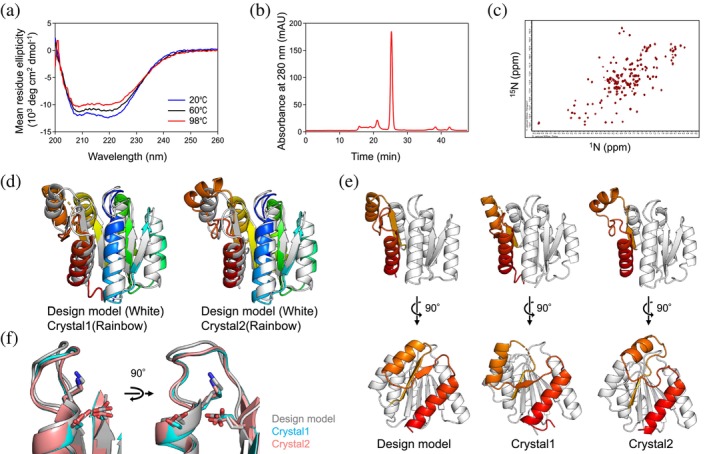
Experimental characterization of the designed protein, PL2x4_2. (a) Far‐ultraviolet CD spectra at temperatures, 20, 60, and 98°C. (b) UV signal from the SEC‐MALS measurement. (c) Two‐dimensional ^1^H–^15^N HSQC NMR spectra at 25°C and 600 MHz. (d, e) Comparison of the design and its crystal structures. The crystal structures reveal two molecules (Crystal 1 and 2) in an asymmetric unit, demonstrating different structural states. The C_α_ RMSD values between the design model and Crystal 1, and the design model and Crystal2, are 1.8 and 1.7 Å, respectively. (f) Close‐up view of the design and crystal structures focusing on the P‐loop motif.

All designed proteins were found to be expressed and highly soluble, and exhibited CD spectra typical of αβ‐proteins from 20 to 98°C (Figures 3a and S1). Two of the three designs, PL2x4_1 and PL2x4_2, were found to be in a monomeric state in solution by SEC‐MALS (Figures 3b and S1). PL2x4_2, which was more soluble than PL2x4_1, displayed well‐dispersed sharp NMR peaks, indicating the folding of the design into rigid tertiary conformations (Figure 3c). Accordingly, we attempted to crystallize the design, PL2x4_2, and determined the structure at 2.4 Å resolution (PDB 9JIX). The crystallographic data revealed two molecules in an asymmetric unit. Each monomer shows a slightly different structural conformation, referred to as Crystal1 and Crystal2 (Figure 3d). Comparison of the designed model with these crystal structures demonstrated good agreement between the designed and experimental structures, except for the fifth and sixth helices, the fifth and sixth strands, and the loops connecting them (Figure 3e). The C_α_ RMSD values between the design model and each monomer in the crystal structure are 1.8 and 1.7 Å, respectively. The values for the C‐terminal structures are 2.3 and 2.8 Å, and those for the other parts are 1.5 and 1.7 Å, respectively. Moreover, the P‐loop motif in the structure exhibited the typical features of the P‐loop motif (Figure 3f). Extra densities near the P‐loop motif suggested the possible presence of a bound sulfate ion, likely due to the ammonium sulfate in the crystallization condition.

**FIGURE 4:**
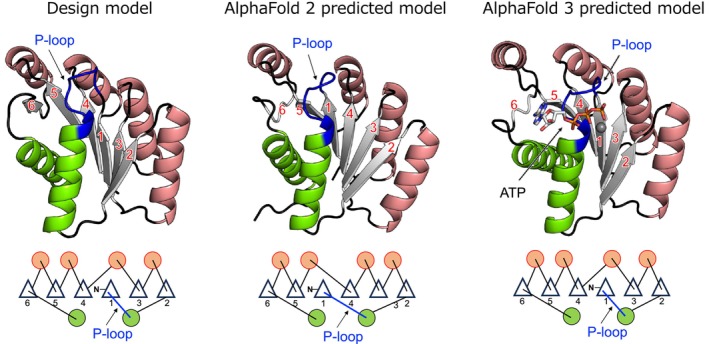
Comparison of the design model (left) with the AlphaFold 2 (middle) and AlphaFold 3 (right) predicted models. The structure and topology of our design model (left) and those of the AlphaFold2 (middle) and AlphaFold 3 (right) predicted models are shown. The order of β‐strands in the AlphaFold 2 predicted model differs from that of the design model: the β‐(P‐loop)‐α‐β motifs in the design and AlphaFold 2 predicted models traverse one and two intervening β‐strands, respectively. The AlphaFold 3 predicted model has the same β‐strand order as the design model.

We predicted the folded structure of the designed protein PL2x4_2 using AlphaFold 2 (Jumper et al., 2021). Interestingly, the predicted structure by AlphaFold 2 exhibited a different topology from the designed structure, which was confirmed to be correct by x‐ray crystallography, specifically in the order of β‐strands (Figures 4 and S2). Prior to the development of AlphaFold, when predicting the folded structures of de novo designed proteins by Rosetta (Rohl et al., 2004), local structures such as supersecondary structures were accurately predicted, but for larger proteins, the prediction of nonlocal structures, particularly the β‐strand order within a β‐sheet, sometimes failed (Koga et al., 2021). We observed the same trend in the AlphaFold 2 prediction. No homologs of our designed protein are present in naturally occurring proteins. Given that AlphaFold is primarily trained on naturally occurring proteins, the absence of homologs posed a challenge for accurately predicting the folded structure. Note that AlphaFold 3 (Abramson et al., 2024) predicted our design model with the correct β‐strand order (Figures 4 and S2). The prediction accuracy for de novo designed proteins might have been improved by learning information from created novel proteins after the development of AlphaFold 2.

**FIGURE 5:**
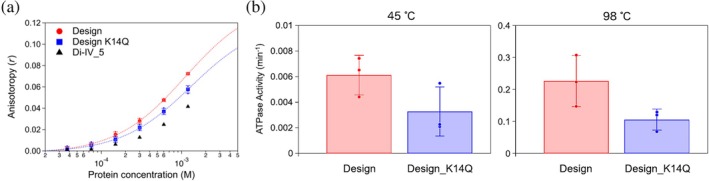
ADP binding and ATP hydrolysis activities of the designed protein, PL2x4_2. (a) ADP binding assays measured by fluorescence polarization for PL2x4_2 (red) and its K14Q mutant (blue). For reference, signals for Di‐IV_5 (Koga et al., 2012), a similar fold protein that was previously designed de novo without the P‐loop motif, are also shown (black). (b) ATP hydrolysis assays conducted using the ATPase/GTPase Activity Assay Kit (Sigma‐Aldrich Co. LLC, MAK113) for PL2x4_2 (red) and the K14Q mutant (blue) at temperatures 45 and 98°C.

Next, the binding affinity of PL2x4_2 for ADP was measured using the fluorescence polarization method with fluorescent‐labeled ADP (Mant‐ADP) (Figure 5a). Clear binding signals were detected for the design, indicating that ADP likely binds to the design. Moreover, the mutant for the conserved Lys residue, K14Q, in the P‐loop motif exhibited a lower signal than the original design, suggesting that the design binds an ADP molecule in proximity to the P‐loop motif. However, the determination of the exact *K*_*d*_ values was challenging due to the expected low affinity, approximately a few mM; it was difficult to prepare protein samples at sufficiently high concentrations. The approximate *K*_*d*_ values estimated from the current signals were 1.46 ± 0.30 mM for the design and 1.88 ± 0.51 mM for the K14Q mutant. For reference, the *K*_*d*_ value of F_1_‐ATPase from thermophilic *Bacillus* PS3 is 19 ± 1 μM (Yasuda et al., 2001), and the median *K*_*M*_ value of naturally occurring enzymes is approximately 100 μM (Mak & Siegel, 2014).

Finally, we measured ATP hydrolysis ability of the design by detecting the product, phosphate molecules, using the malachite green reagent (Figure 5b). The values for ATP hydrolysis activities were obtained after subtracting signals for ATP solutions in the absence of protein as the background (Figure S3). The design exhibited ATP hydrolysis activity with a hydrolysis rate of approximately 0.0061 ± 0.0015 min^−1^ at 45°C. Moreover, consistent with the binding affinity measurements, the K14Q mutant shows decreased ATPase activity; the rate of ATP hydrolysis is approximately 0.0033 ± 0.0019 min^−1^ at 45°C. These results demonstrate that the designed protein with the P‐loop motif has catalytic ability. However, the observed activity was comparable to those of previously designed proteins (Romero Romero et al., 2018; Wang & Hecht, 2020): the ATP hydrolysis rate of the ATPase designed by Romero et al. is 0.0058 ± 0.0012 min^−1^ at 45°C (Romero Romero et al., 2018) and the *k*_*cat*_ value of the ATPase designed by Wang et al. is 0.00058 ± 0.00005 min^−1^ at room temperature (Wang & Hecht, 2020). To further investigate, we tested the activity of the design at a higher temperature (98°C). At this temperature, the hydrolysis activity drastically increased to approximately 0.23 ± 0.08 min^−1^ (that of the K14Q mutant is approximately 0.11 ± 0.03 min^−1^). This increase in activity can be attributed to the high‐temperature stability of the design, although its activity levels still fall short compared to those of naturally occurring enzymes. For reference, the *V*_max_ value of F_1_‐ATPase from thermophilic *Bacillus* PS3 is 247 ± 9 s^−1^ at 23°C (Yasuda et al., 2001), and the median *k*_*cat*_ values of naturally occurring enzymes is about 1.0 × 10^2^ s^−1^ (Mak & Siegel, 2014).

## 3 DISCUSSION

We aimed to design an ATPase incorporating the P‐loop motif by investigating the optimal backbone blueprint to harbor the P‐loop motif in designed structures. To achieve this, we conducted a statistical analysis of naturally occurring proteins with the P‐loop motif and performed Rosetta folding simulations. Based on the identified backbone blueprint, we designed three proteins, two of which successfully folded into monomeric structures with high thermal stability. The crystal structure of one of these designs, PL2x4_2, was determined and found to be nearly identical to the design model, both in overall structure and in the P‐loop motif. In previous work by Romero et al., the P‐loop motif was incorporated into the β‐α‐β motif, where the first and second β‐strands are adjacent to each other (Romero Romero et al., 2018). The resulting P‐loop motif shows unstable and dynamical conformations in the solved NMR structures (PDB 6C2U and 6C2V), unlike the P‐loop motif observed in naturally occurring proteins. We identified characteristic features of the β‐(P‐loop)‐α‐β motif: the number of β‐strands traversed by the motif is greater than zero. Incorporating this feature into the blueprint for de novo design, along with the optimization of geometries and lengths of other secondary structures and loops, likely enabled the successful de novo designed structure harboring the P‐loop motif with its ideal features (Figure 3).

**FIGURE 6:**
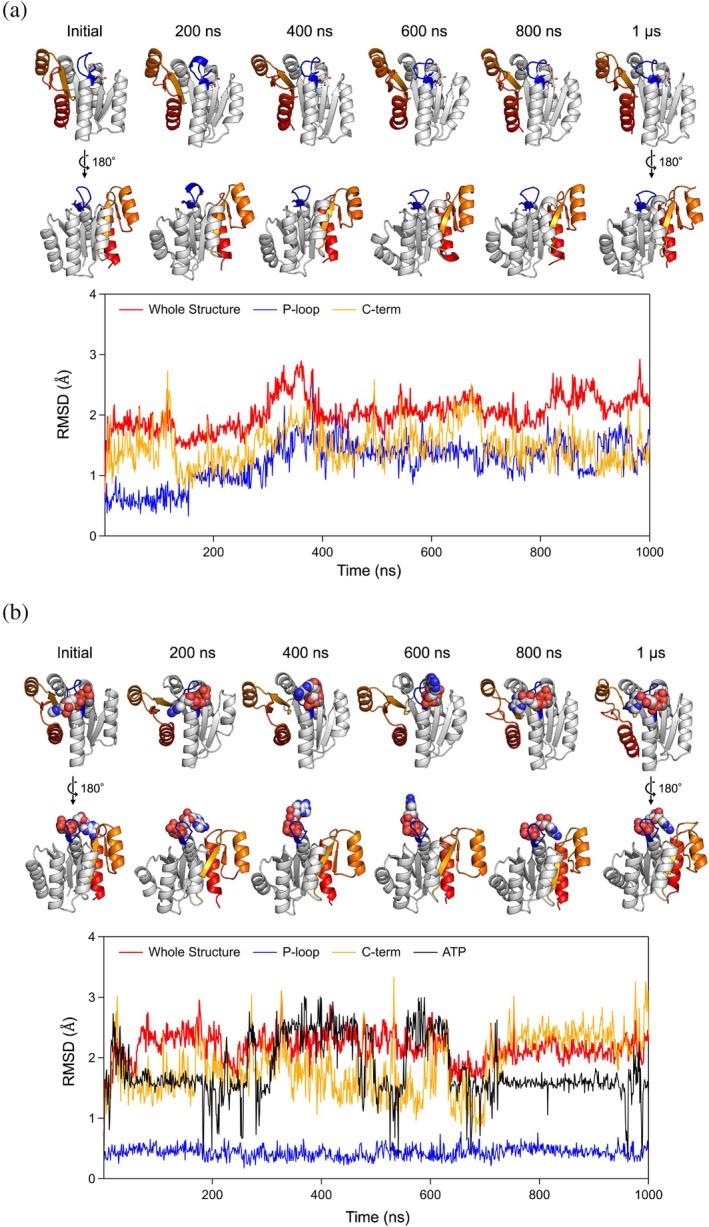
The created binding pocket for adenine of ATP may not be optimal. (a) MD simulation of the designed PL2x4_2. (b) MD simulation of the designed PL2x4_2 complexed with an ATP molecule. Top: structures at the initial state, 200 ns, 400 ns, 600 ns, 800 ns, and 1.0 μs from the MD trajectory are shown. Bottom: the RMSD values for the entire structure (red), the P‐loop motif (blue), the C‐terminal structure (orange) and ATP molecule (black) are shown. In the complex, while the P‐loop motif maintains its initial conformation during the simulation, the C‐terminal structure and the ATP molecule undergo conformational changes. The adenine ring of the simulated ATP molecule (sphere) was dissociated from the designed binding pocket. The pocket collapsed due to structural changes in the C‐terminal region (colored region in the protein structure).

The de novo designed protein with the ideal P‐loop motif demonstrated ADP binding and ATP hydrolysis abilities as expected (Figure 5). However, these abilities are lower than those of typical naturally occurring ATPases. To investigate the reasons for this reduced functionality, we conducted three independent 1.0 μs MD simulations each for the designed protein both in its unbound state and in complex with an ATP molecule and a magnesium ion (Figures 6 and S4). The simulations suggested that the low binding and hydrolysis activities might be due to a suboptimal binding pocket for the adenine and ribose moieties of ATP, as these moieties were detached from the binding pocket during the simulations. Furthermore, we found that the loop (the C‐terminal loop connected the sixth strand) adjacent to the binding pocket exhibited unsteady dynamic motion. Interestingly, in simulations where the P‐loop motif was stabilized by interaction with the phosphate in the ATP‐complex state, the dynamical motion of the C‐terminal structure was significantly greater than in the unbound state. This motion could contribute to the weak binding of the pocket against the adenine and ribose moieties. Consistent with the simulations, each monomer in our crystal structure revealed different loop conformations, suggesting that the loop's flexibility might indeed contribute to the suboptimal binding pocket. Note that the AlphaFold 3 prediction models for the complex state with an ATP molecule supported the binding of ATP to our designed protein but did not predict the dynamic features of the C‐terminal structure.

We successfully designed a protein that accommodates the P‐loop motif in its ideal conformation. However, the ADP binding and ATP hydrolysis activities were not as high as typically naturally occurring ATPases. Detailed analysis of the de novo designed protein using MD simulations suggests that the binding pocket for the adenine and ribose moieties of ATP is not optimal, indicating that the P‐loop motif alone is insufficient to achieve high enzymatic activity. Additionally, the absence of the Arg‐finger, which is known to be a crucial component of the ATP hydrolysis module (Komoriya et al., 2012) that works with the P‐loop and Walker‐B motifs, would contribute to the low activity. Moreover, protein dynamics that facilitate the catalytic activity must be taken into account (Kosugi & Hayashi, 2012). The blueprint developed in this work was optimized for folding ability, but longer loops or local frustrations encoding functional dynamics can be important to achieve high ATPase activity. Future investigations should focus on designing blueprints and amino acid sequences that include these features.

## 4 MATERIALS AND METHODS

### 4.1 Backbone building and sequence design protocol

The backbone structures for the blueprint were built by Rosetta sequence‐independent folding simulations using coarse‐grained model structures, in which each residue is represented by main chain atoms (N, H, CA, C, and O) and a side‐chain pseudo atom (Rohl et al., 2004). The following terms were included in the Rosetta potential function used in the simulations with no sequence‐dependent score terms: steric repulsion (vdw = 1.0), overall compaction (rg = 1.0), and hydrogen bonds (hbond_sr_bb = 1.0, hbond_lr_bb = 1.0). The steric residues of Val were used for that of the side‐chain pseudo atom. An ATP molecule for each generated main‐chain structure was placed near the P‐loop motif using the Rosetta PredesignPerturb protocol, which randomly perturbs a ligand in a protein active site, with the distance constraints used in our previous research (Kosugi et al., 2023). Sequence design was performed by RosettaDesign calculation (Leaver‐Fay et al., 2011) using the flexible‐backbone design (FlxbbDesign) protocol with the full‐atom Talaris2014 scoring function (O'Meara et al., 2015). The definition of the environment for each residue position, layer, and the designated amino acids for each layer are almost the same as in the previous paper (Koga et al., 2012), except for the point that we excluded large aromatic residues, F and W, from the boundary layer in this paper. We also added constraints for the strand pairs to keep the backbone structure. The parameters for ATP‐Mg^2+^ were determined using those for the corresponding atom types defined in Rosetta. A set of various ATP conformations was generated by BCL software (Kothiwale et al., 2015).

### 4.2 Molecular dynamics (MD) simulations

The AMBER14 (Case et al., 2014) (first screening) and AMBER22 (Case et al., 2022) (evaluation of ATP binding) software suite were used for MD simulations. The design models were used as the initial structures, to which hydrogen atoms were added by the LEaP module of AMBER14. The simulation system contains a designed monomer with ATP placed in a water box of approximately 65 Å × 75 Å × 65 Å. To neutralize the system, 1 or 2 sodium ions or 2 chloride ions were put in the box. AMBER ff99SB (first screening) or ff14SB (evaluation of ATP binding) sets and TIP3P were utilized for the protein and water molecules, respectively. Parameters for the ATP molecule were adopted from a reference paper (Meagher et al., 2003). Long‐range electrostatic interactions were treated by the particle mesh Ewald (PME) method. Non‐bonded interactions were cut off at 10 Å. After carrying out a short minimization to remove artificial repulsions in the initial structure, 100 ns (first screening) and 1.0 μs (evaluation of ATP binding) MD simulations in a constant‐NPT (300 K, 1 atm) ensemble were performed after the 100 ps heating stage with the NVT ensemble (the time step is 2.0 fs and hydrogen atoms were constrained with SHAKE procedure). At the heating step, the temperature was raised gradually from 0 to 300 K with weak restraints (10 kcal/mol/A^2^) on the atoms of the designed protein.

### 4.3 Plasmid construction, expression, and purification of designed proteins

The genes encoding the designed sequences (Table S1) were synthesized and cloned into pET21b vectors (Eurofins Genomics). *E. Coli* BL21* (DE3) competent cells were transformed with the plasmid and cultured at 37°C for 6 h followed by overnight incubation at 18°C in autoinduction media (Studier, 2005). The expressed proteins were purified using a Ni‐NTA column and then dialyzed against Tris buffer (10 mM Tris–HCl (pH 8.0), 100 mM NaCl and 5 mM MgCl_2_). The purified protein samples were stored at −80°C. For the ATP‐binding and ATPase activity assay, the samples were concentrated with a Vivaspin20 5000 molecular weight cutoff (MWCO) (Cytiva) and then passed through a Superdex 75 Increase column (Cytiva) equilibrated with Tris buffer (pH 7.4). The purified samples were stored at −80°C.

### 4.4 Plasmid construction for the design mutants

Mutations were introduced by the Quick Change Multi Site‐Directed Mutagenesis Kit from Agilent Technologies. The expression and purification for the mutants were carried out by the same protocol as for the original design. The DNA sequence was confirmed by DNA sequencing analysis performed by Fasmac.

### 4.5 Circular dichroism (CD) measurement

All CD measurements were performed using a JASCO J‐1500 CD spectrometer, using a 1 mm path length cuvette. Far‐UV CD spectra for the designed proteins were measured in the wavelength range from 260 to 200 nm. The measurements were conducted at various temperatures ranging from 25 to 98°C for protein samples at concentrations of 10–50 μM in Tris buffer (pH 8.0).

### 4.6 SEC‐MALS

SEC‐MALS experiments were performed using a miniDAWN TREOS static light scattering detection system from Wyatt Technology Corporation, combined with an HPLC system (1260 Infinity LC; Agilent Technologies) with a Superdex 75 increase 10/300 GL column from Cytiva. After equilibration of the column with PBS buffer (pH 7.4), the volume of 100 μL of protein samples (300–500 μM) in PBS buffer (pH 7.4), which were purified beforehand by Ni‐affinity columns, was injected. Protein concentrations were calculated from the absorbance at 280 nm detected by the HPLC system. Static light scattering data were collected at three different angles, 43.6°, 90.0°, and 136.4°, using a 659 nm laser. These data were analyzed by the ASTRA software (version 6.1.2, Wyatt Technology Corporation) with a change in the refractive index with concentration, a *dn/dc* value of 0.185 mL/g to estimate the molecular weights of the dominant peaks.

### 4.7 Expression and purification of protein sample for NMR measurements

The designed proteins were expressed in *E. Coli* BL21* (DE3) cells as uniformly ^15^N‐labeled proteins. These ^15^N‐labeled proteins were expressed by using MJ9 minimal media, which contains ^15^N ammonium sulfate as the sole nitrogen source and ^12^C glucose as the sole carbon source. The expressed proteins with a 6xHis tag were purified using a Ni‐NTA column and then dialyzed to Tris buffer (10 mM Tris–HCl (pH 8.0), 100 mM NaCl and 5 mM MgCl_2_). The samples were concentrated with a Vivaspin20 5000 molecular weight cutoff (MWCO) (Cytiva) and then passed through a Superdex 75 Increase column (Cytiva) equilibrated with PBS buffer (pH 7.4). The purified samples were stored at −80°C.

### 4.8 ^1^H– ^15^N HSQC NMR spectroscopy

To confirm the core packing of the designed proteins, we measured 2D ^1^H–^15^N HSQC spectra for the most promising design. The spectra were collected for 400–500 μM samples in 90% ^1^H_2_O/10% ^2^H_2_O PBS buffer (pH 7.4) at 25°C on a JEOL JNM‐ECA 600 MHz spectrometer and were processed and analyzed using JEOL Delta NMR software.

### 4.9 Expression and purification of protein sample for crystallization

The protein sample for crystallization was prepared by removing the His‐tag from the N‐terminal of the designed protein. The gene encoding the designed PL2x4_2 sequence was cloned into the pET21b vector. A TEV protease cleavage site and a Gly residue were inserted between the gene encoding the designed protein and the His‐tag in the pET15b vector. Using this plasmid, the designed protein was expressed and subsequently purified via a Ni‐NTA column. The eluted sample was mixed with TEV protease in a 10:1 molar ratio and dialyzed against a 20 mM Tris–HCl buffer (pH 8.0). These dialyzed samples was applied to a Ni‐NTA column, and the flow‐through was collected. Next, the sample was loaded onto a HiTrap Q HP column (Cytiva) equilibrated with 20 mM Tris–HCl buffer (pH 8.0) and then eluted with a linear gradient of 20 mM Tris–HCl buffer with 0–1000 mM NaCl in 20 min at a flow rate of 1.0 mL / min^−1^. The concentrated sample with a Vivaspin 20 (5000 MWCO) (Sartorius) was loaded onto a Superdex 75 Increase 10/300 GL column (Cytiva) equilibrated with Tris buffer without MgCl_2_ (10 mM Tris–HCl (pH 8.0) and 100 mM NaCl) at a flow rate of 0.5 mL / min^−1^. The purified sample was concentrated using a Vivaspin 500 (5000 MWCO).

### 4.10 Crystallization, data collection, and structure determination

The sitting drop vapor diffusion method was used for crystallization. Crystals of PL2x4_2 were obtained by mixing a 2.0 μL protein solution (37.7 mg/mL protein in Tris buffer without MgCl_2_) with 2.0 μL of the reservoir solution (0.1 M Sodium Acetate (pH 4.5) and 2.0 M Ammonium Sulfate), which was equilibrated against 500 μL of reservoir solution. The crystals appeared in 1–2 weeks at a temperature of 293 K. The reservoir solution with 40% (v/v) concentration of glycerol was gradually added to the drop containing crystals up to a 20% (v/v) concentration.

The crystals were mounted on cryo‐loops (Hampton Research), flash‐cooled, and stored in liquid nitrogen for preservation. X‐ray diffraction data were collected using a wavelength of 1.1 Å on BL‐1A of the Photon Factory (PF) using a single crystal at the cryogenic temperature of 100 K. The collected data were processed by XDS (Kabsch, 2010). The structure was determined by the molecular replacement method with Phaser (McCoy et al., 2007), using the designed model as an initial search template. The model was iteratively refined with PHENIX (Adams et al., 2010) and REFMAC5 from the CCP4 Suite (Murshudov et al., 1997) and manually corrected using COOT (Emsley et al., 2010). The figures in the manuscript were generated by PyMOL (Schrodinger, LLC, 2018). The crystallographic and refinement statistics are summarized in Table S2. The crystal structures have been deposited in the wwPDB as PDB 9JIX.

### 4.11 Fluorescence polarization measurement for evaluating ADP‐binding affinity

Fluorescence polarization‐based affinity measurements for the designed proteins were performed using 1 μM fluorescent‐labeled ADP, Mant‐ADP. We observed changes in fluorescence anisotropy (*r*) of the fluorescent‐labeled ADP mixed with the protein samples at varying concentrations. The mixtures were prepared in Greiner black flat bottom 96‐well plates and equilibrated for 1 h at room temperature. Measurements were carried out on a Spark 10 M (TECAN), using 360 nm excitation and 464 nm emission, each with a 35 nm bandwidth. All measurements were conducted in Tris buffer.

Equilibrium dissociation constants (*K*_*d*_) were determined by fitting the observed anisotropy data, which was obtained from 20 measurements over a 10‐min period for each protein concentration, to Equation (1). In this equation, *A* is experimentally measured anisotropy, *A*_*f*_ is anisotropy of the free ligand, *A*_*b*_ is the anisotropy of the fully bound ligand, [*L*]_*T*_ is the total ligand concentration, and [*R*]_*T*_ is the total protein concentration. The *K*_*d*_ values were determined by averaging the values from three independent measurements.

(1) $$A=A_{f}+\left(A_{b}-A_{f}\right)\times \left(\frac{\left({\left[L\right]}_{T}+K_{d}+{\left[R\right]}_{T}\right)-\sqrt{{\left(-{\left[L\right]}_{T}-K_{d}-{\left[R\right]}_{T}\right)}^{2}-4{\left[L\right]}_{T}{\left[R\right]}_{T}}}{2{\left[L\right]}_{T}}\right).$$

### 4.12 ATPase activity measurements

ATPase activities for the designed proteins were measured by the ATPase/GTPase Activity Assay Kit (Sigma‐Aldrich Co. LLC, MAK113). Protein samples at concentrations of 25 μM (for reactions at 45°C) or 5 μM (for reactions at 98°C) were incubated with 1 mM ATP in 40 μL Assay Buffer (40 mM Tris, 80 mM NaCl, 8 mM MgAc_2_, 1 mM EDTA, pH 7.5) in 96 well plates (Greiner, 665801) for 30 min at 45°C or 5 min at 98°C, respectively. As a background reference, solutions in the absence of protein were also incubated under the same conditions. After incubation, the samples were immediately put on ice, and 200 μL of malachite green reagent was added to each well, and the mixtures were incubated for 30 min at room temperature. The absorbance of the incubated solutions was measured between 350 and 850 nm using a Spark 10 M (TECAN). The relative absorbances, as signals from only enzymatic activity, were calculated by subtracting the background signal from ATP hydrolysis in the absence of protein because the background is quite high, as shown in Figure S3. The product (phosphate) concentration was estimated from the relative absorbance at 620 nm, using the reference solution for comparison against standard buffers at several known phosphate concentrations. ATPase activities for the designed proteins were calculated from the estimated product concentrations.

*Supporting information description*: Supporting Information is available for this paper. The designed model structure will be provided as Supporting Information.

## Supporting information


**Figure S1.** Characterization of all designs.
**Figure S2.** AlphaFold 2 and 3 predicted structure models colored by pLDDT values.
**Figure S3.** Raw signals of ATP hydrolysis assay at 98°C.
**Figure S4.** MD simulations of designed protein, PL2x4_2.
**Table S1.** Amino acid sequences of designed proteins.
**Table S2.** Data collection and refinement statistics of crystal structure.

## Data Availability

The data that support the findings of this study are available from the corresponding author upon reasonable request.
