# Supplementary material for: De novo design of ATPase based on a blueprint optimized for harboring the P‐loop motif
Source: Protein Sci. 2025 May 13;34(6):e70132. doi: 10.1002/pro.70132 (PMC12075096; doi:10.1002/pro.70132)
Supplement: Supplementary file 1 — Figure S1. Characterization of all designs. Figure S2. AlphaFold 2 and 3 predicted structure models colored by pLDDT values. Figure S3. Raw signals of ATP hydrolysis assay at 98°C. Figure S4. MD simulations of designed protein, PL2x4_2. Table S1. Amino acid sequences of designed proteins. Table S2. Data collection and refinement statistics of crystal structure. [file PRO-34-e70132-s001.pdf]

Supporting Information

De novo design of ATPase based on a blueprint optimized for harboring the P-loop motif

Takahiro Kosugi[1,2,3,4*], *Mikio Tanabe*[5], *Nobuyasu Koga*[1,2,3,6*]

[1]Research Center of Integrative Molecular Systems, Institute for Molecular Science (IMS), National Institutes of Natural Sciences (NINS), Okazaki, Aichi, 444-8585, Japan

[2]Exploratory Research Center on Life and Living Systems (ExCELLS), National Institutes of Natural Sciences (NINS), Okazaki, Aichi, 444-8585, Japan

[3]Molecular Science Program, SOKENDAI (The Graduate University for Advanced Studies), Hayama, Kanagawa, 240-0193, Japan

[4]PRESTO, Japan Science and Technology Agency, Kawaguchi, Saitama 332-0012, Japan

[5]Structural Biology Research Center, Institute of Materials Structure Science, High Energy Accelerator Research Organization (KEK), Tsukuba, Japan

[6]Advanced Data Science Center for Protein Research (ASPiRE), Institute for Protein Research (IPR), Osaka University, Suita, Osaka 565-0871, Japan

*To whom correspondence should be addressed. E-mail: takahirokosugi@ims.ac.jp or nkoga@protein.osaka-u.ac.jp

Table of Contents

Figure S1: Characterization of all designs

Figure S2: AlphaFold 2 and 3 predicted structure models colored by pLDDT values

Figure S3: Raw signals of ATP hydrolysis assay at 98 °C

Figure S4: MD simulations of designed protein, PL2x4_2

Table S1: Amino acid sequences of designed proteins

Table S2: Data collection and refinement statistics of crystal structure

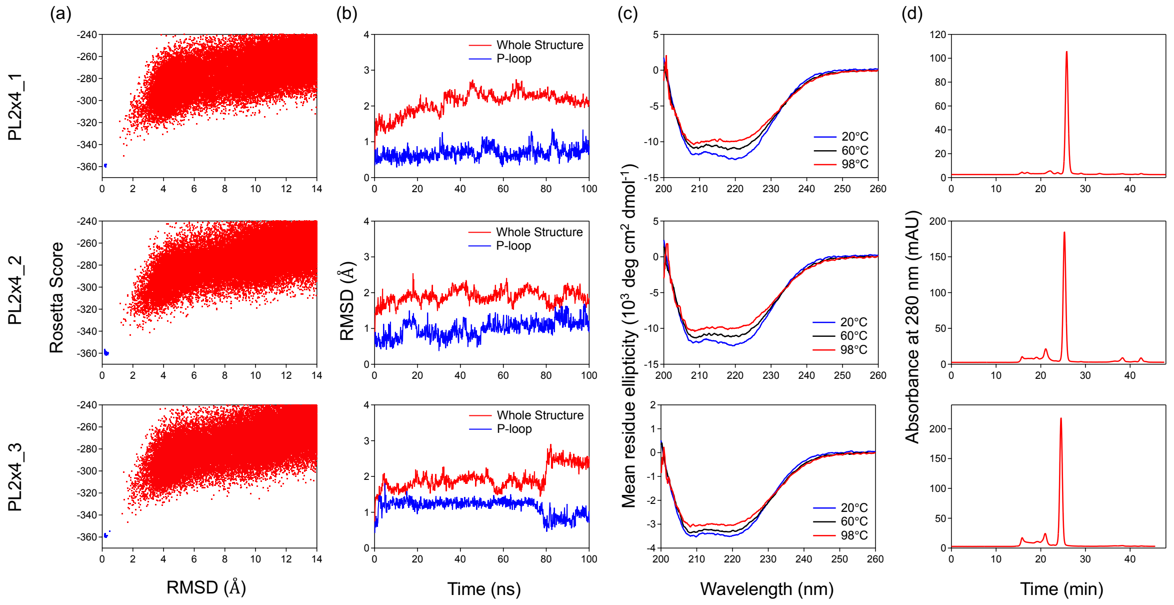

Figure S1. Characterization of all designs

(a) Energy landscapes from Rosetta ab initio structure prediction simulations. (b) Cα root mean square deviation (RMSD) values during MD simulations for designs without ATP. (c) Far-ultraviolet circular dichroism (CD) spectra at various temperatures. (d) UV signals from size-exclusion chromatography combined with multi-angle light scattering (SEC-MALS).

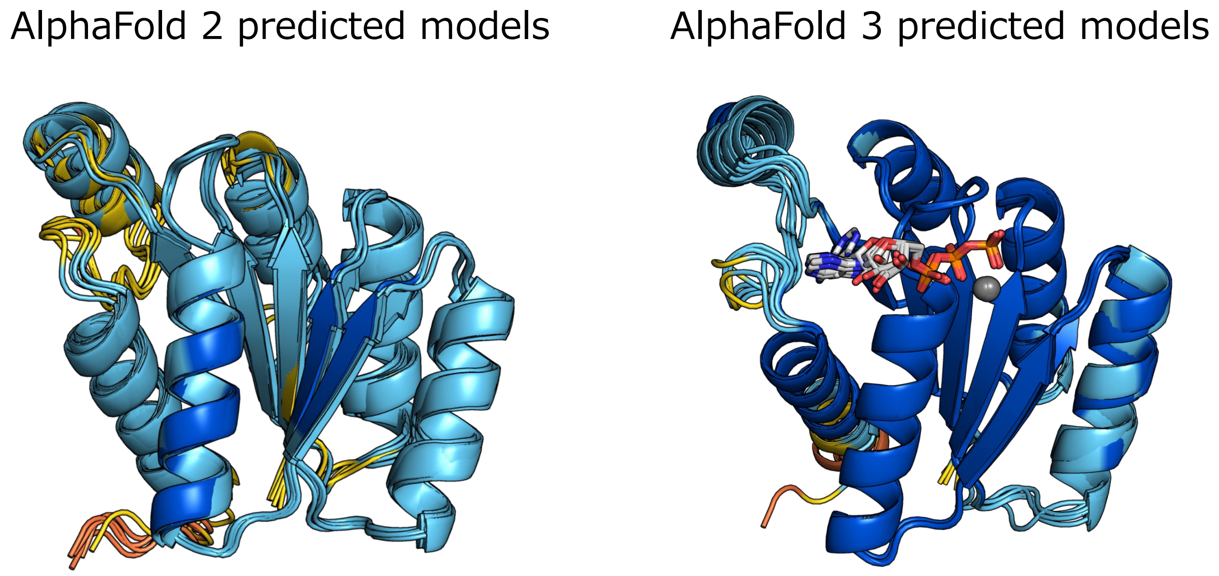

Figure S2. AlphaFold 2 and 3 predicted structure models colored by pLDDT values

Five structures predicted by AlphaFold 2 (left) and 3 (right) were superimposed and colored by pLDDT values. All predicted structures are similar and exhibit high overall pLDDT scores.

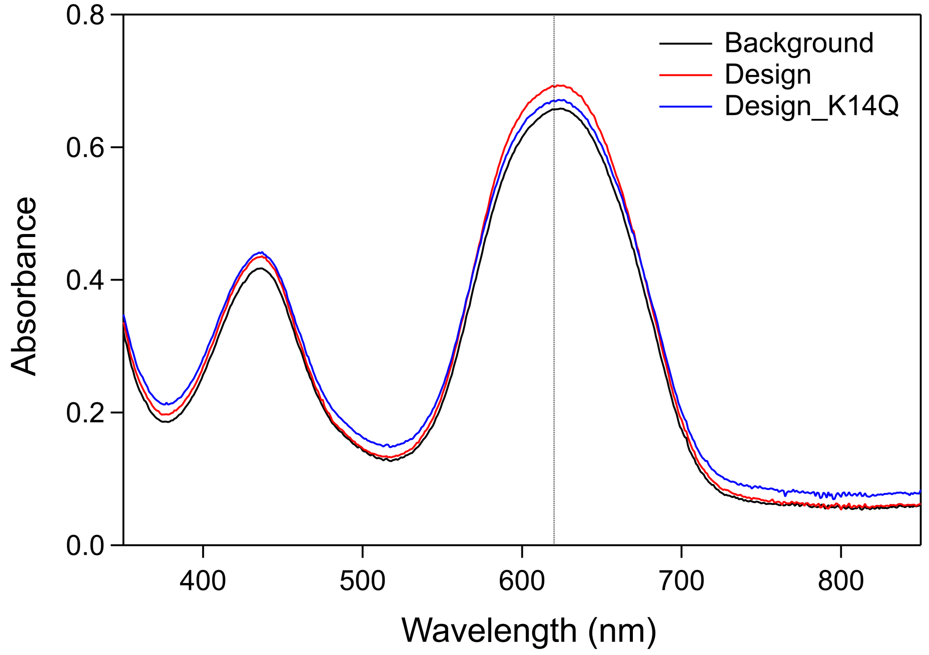

Figure S3. Raw signals of ATP hydrolysis assay at 98 °C

Raw signals for the background (black), ATP solution in the absence of protein samples, the designed protein (red), and the K14Q mutant (blue) are shown. The values obtained after subtracting the background signal at 620 nm were used to calculate the ATPase activity.

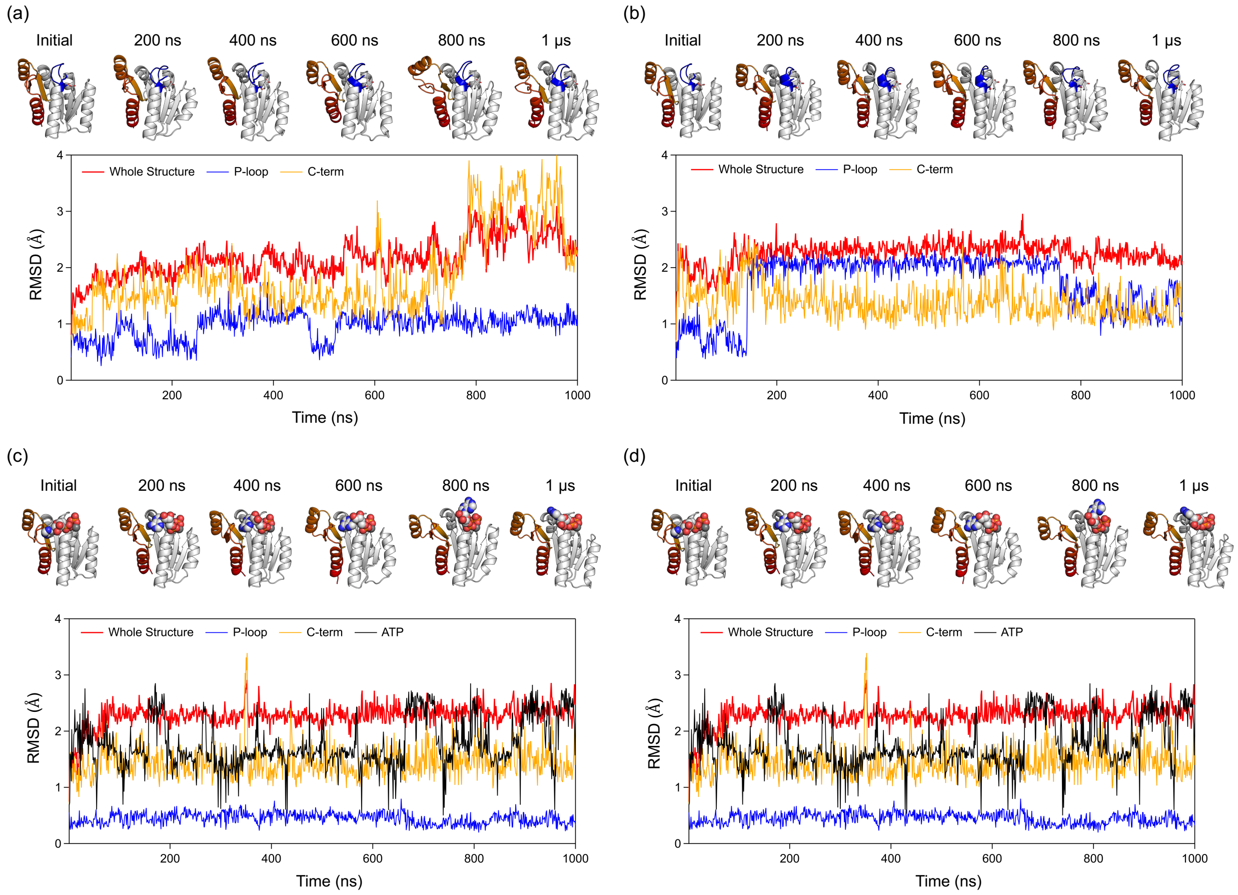

Figure S4. MD simulations of designed protein, PL2x4_2

MD simulations for the designed protein PL2x4_2 in the unbound state (a, b) and in the complex with an ATP molecule (c, d). Top: Structures at the initial state, 200 ns, 400 ns, 600 ns, 800 ns and 1.0 μs from the MD trajectories are shown. Bottom: the RMSD values for the entire structure (red), the P-loop motif (blue), the C-terminal structure (orange) and ATP molecule (black) are plotted.

Table S1:Amino acid sequences of designed proteins

Computationally designed amino acid sequences are shown in uppercase and amino acid residues added to allow expression, purification, concentration measurement, cleavage sites of restriction enzymes and the spacer between the designed sequence and the C-terminal His-tag are shown in lowercase.

Name	Amino acid sequence
**PL2x4_1**	mgAIVILVVGPPGSGKSQLIEAIERLARKQGQPVVTTSVTSEDEAKKVLRHLLKRD<br>PNAIVVIEIKSPSIAERVAEEVLRQDPTAVLVVVVSSPDQARKLREQLPNVIVVVLI<br>RDPEKLKEAKKEGTQVLSGNGNPEEAAKIIAQLIKDQAgswslehhhhhh
**PL2x4_2**	mgAIVILVVGPPGSGKSQLIKAIEKLAREQGQPVVTTSVTSEDEAKKVLEELLKKDP<br>NAIVVIEIKNPRIAERVAKRVLEEDPTAVLVVVVSSPEVARELRENLPNVIVVVLIR<br>DPEKLKEAKKQGTQVLSGDGNPEEAAKQIAQLIKDQAgswslehhhhhh
**PL2x4_3**	mgAIVILVVGPPGSGKSQLIKAIEKLAREQGQPVITTSVTSEDEAKEELERLLKKDP<br>NAIVVIEIKSSRIAERVAKRVWEEDPTAVLVVVVSSPEDARELRENLPDVIVVVLIR<br>DPEKLKEAKKEGTQVLSGNGNPEEAAKIIAQLIKDQAgslehhhhhh

Table S2: Data collection and refinement statistics of crystal structure

	**PL2x4_2 PDB: 9JIX**
**Data collection**	
**Space group**	$P3_121$
**Cell dimensions**	
a, b, c **(Å)**	77.66, 77.66, 101.54
**α, β, γ (°)**	90.0, 90.0, 120.0
**Wavelength**	1.070
**Resolution (Å)**	40.52 – 2.29 (2.37 – 2.29)
R_{merge}	0.059 (1.012)
I/σI	18.3 (2.3)
$C/C_{1/2}$	1.000 (0.785)
**Completeness (%)**	100.0 (100.0)
**Redundancy**	9.8 (10.4)
**Refinement**	
**Resolution (Å)**	40.52 - 2.29
**No. reflections**	16412
R_{work}/R_{free}	0.237/0.260
**No. atoms**	
**Protein**	2308
**Ligand/ion**	31
**Water**	19
**B-factors**	
**Protein**	79.4
**Ligand/ion**	90.0
**Water**	57.8
**R.m.s. deviations**	
**Bond length (Å)**	0.011
**Bond angles (°)**	1.602

A single crystal was used to obtain data set. Values in parentheses are for highest-resolution shell.
